# A Rare Cause of Headache and an Unorthodox Transfer: A Case Report

**DOI:** 10.5811/cpcem.2022.10.57491

**Published:** 2023-01-11

**Authors:** Samuel L. Burleson, Joe Butler, Gabrielle Gostigian, Matthew S. Parr, Matthew P. Kelly

**Affiliations:** * University of Alabama at Birmingham, Department of Emergency Medicine, Birmingham, Alabama; † Baptist Memorial Hospital – Golden Triangle, Department of Emergency Medicine, Columbus, Mississippi; ‡ University of Alabama at Birmingham, Department of Neurosurgery, Birmingham, Alabama

**Keywords:** boarding, transfer, colloid cyst, intracranial hypertension, case report

## Abstract

**Introduction:**

Emergency department (ED) crowding and hospital diversion times are increasing nationwide, with negative effects on patient safety and an association with increased mortality. Crowding in referral centers makes transfer of complex or critical patients by rural emergency physicians (EP) more complicated and difficult. We present a case requiring an unorthodox transfer method to navigate extensive hospital diversion and obtain life-saving neurosurgical care.

**Case Report:**

We present the case of a previously healthy 21-year-old male with two hours of headache and rapid neurologic decompensation en route to and at the ED. Computed tomography revealed obstructive hydrocephalus recognized by the EP, who medically managed the increased intracranial pressure (ICP) and began the transfer process for neurosurgical evaluation and management. After refusal by six referral centers in multiple states, all of which were on diversion, the EP initiated an unorthodox transfer procedure to the institution at which he trained, ultimately transferring the patient by air. Bilateral external ventricular drains were placed in the receiving ED, and the patient ultimately underwent neurosurgical resection of an obstructive colloid cyst.

**Conclusion:**

First, our case illustrates the difficulties faced by rural EPs when attempting to transfer critical patients when large referral centers are refusing transfers and the need for improvements in facilitating timely transfers of critically ill, time-sensitive patients. Second, EPs should be aware of colloid cysts as a rare but potentially catastrophic cause of rapid neurologic decline due to increased ICP, and the ED management thereof, which we review.

## INTRODUCTION

Nationwide, there has been a significant increase in hospital diversion times and emergency department (ED) crowding that has direct negative effects on patient safety, health, and quality of service and is associated with increased mortality.^1^ Crowding is also associated with delays in time-sensitive treatments, increased preventable errors,^2^ poor compliance with recommended care, increased hospital stays and costs, and increased stress for healthcare workers.^3^,^4^ Prior to the coronavirus 2019 (COVID-19) pandemic, ED visits increased over 60% since 1997 to approximately 146 million, with nearly 46 visits per 100 persons in 2016.^5^ Boarding issues have only worsened since the COVID-19 pandemic, while simultaneously increasing overall ED patient lengths of stay.^6^

Boarding and crowding in referral centers increases the difficulties and delays faced by rural emergency physicians (EP) and EDs in transferring complex, often critically ill patients to those centers for definitive care. The COVID-19 pandemic has exacerbated these issues and made the transfer process an even more difficult one. We present a case requiring an unorthodox transfer method to obtain life-saving neurosurgical care.

## CASE REPORT

A 21-year-old male with no past medical history presented to a rural ED with severe headache and decreased level of consciousness. He was brought by his friend, who stated the patient complained of a headache that began two hours prior to arrival while he was working at a local grocery store. En route, he acutely developed slurred speech and became unresponsive in the vehicle. There was no history of trauma, fevers, or illicit drug use.

The patient’s initial heart rate was 137 beats per minute (bpm), blood pressure 159/85 millimeters of mercury (mm Hg), and respiratory rate 17 breaths per minute, with a room-air oxygenation saturation of 100%. The patient was not following commands, but he opened his eyes to pain, and moved all extremities. He did not respond to verbal stimuli. His Glasgow Coma Scale was calculated to be eight. Corneal, gag, and cough reflexes were intact. Pupils were 3 millimeters and reactive bilaterally. Fundoscopic exam was not performed.

**Image 1:**
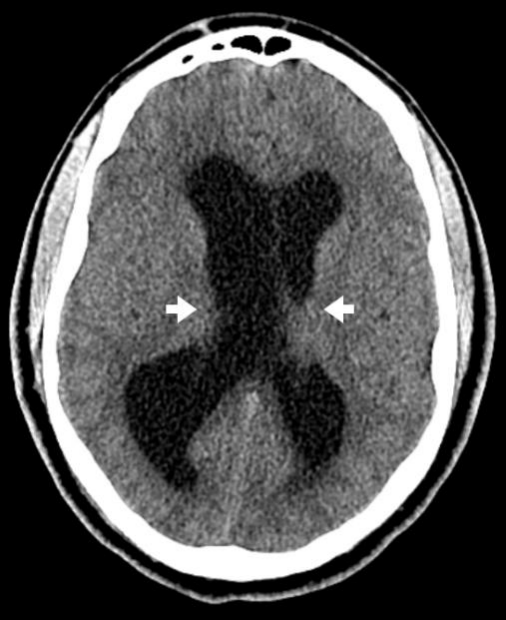
Axial computed tomography revealing enlarged lateral ventricles (white arrows) and hydrocephalus. No dilation of third or fourth ventricles or definitive signs of herniation were noted.

The patient was intubated on arrival for airway protection. Laboratory tests were unremarkable other than a lactic acidosis of 4.8 millimoles/liter (mmol/L) (reference range: 0.0–2.2 mmol/L), increasing to 7.8 mmol/L three hours later. Non-contrasted computed tomography (CT) of the head (Image 1) was obtained and recognized as hydrocephalus by the EP. No neurosurgical service was available at the ED, so the transfer process was initiated. The EP called the transfer centers of six hospitals with neurosurgical services across multiple states, none of which could accept the transfer.

*CPC-EM Capsule*

What do we already know about this clinical entity?

*Colloid cysts are a rare cause of headache causing potentially catastrophic neurologic decompensation and requiring extensive resuscitation and neurosurgical intervention.*

What makes this presentation of disease reportable?

*This case required extensive ED management of increased intracranial pressure due to a colloid cyst, as well as an unorthodox transfer technique in order to obtain definitive therapy.*

What is the major learning point?

*Emergency physicians must be facile in the management of increased intracranial pressure, and creative and persistent in navigating logistical challenges in the era of hospital diversion.*

How might this improve emergency medicine practice?

*We hope our case will educate emergency physicians in the management of increased intracranial pressure, as well as illustrate the need for improvements in the current hospital transfer system.*

After CT, the patient developed decorticate posturing and was administered a bolus of 250 milliliters (mL) of 3% hypertonic saline, the head of his bed was raised to 30 degrees, propofol infusion was increased, and fentanyl infusion was added. The patient’s heart rate increased to 167 bpm, and blood pressure increased to 232/143 mm Hg with the right pupil dilated and unresponsive. A one gram per kilogram bolus of 20% mannitol and additional bolus 250 mL of 3% saline were administered.

After multiple failed attempts at transfer, the EP called the unpublished number of the attending EP workstation at the institution where he had trained, and that institution eventually received the patient and requested assistance. Typically, transfers to the accepting facility are routed through a transfer call center, which contacts the attending physician for the specialty service, who discusses the case with the transferring physician and arranges further care. If the required higher level of service is unavailable, the transfer call center prevents the transfer without input from accepting services. In this case, the transferring EP was unable to communicate directly with the attending neurosurgeon because the neurological intensive care unit (ICU) was full, and the transfer call center followed established protocol and refused the transfer.

The receiving EP and the attending neurosurgeon discussed the time-sensitive, life-threatening nature of the case and the difficulties in transfer. The neurosurgeon accepted the patient to the ED and began treatment there. A neurosurgery resident and a neurologic ICU nurse were made available to treat the patient in the ED while preparations were made to find a neurologic ICU bed. The patient was flown by helicopter to the accepting facility.

**Image 2:**
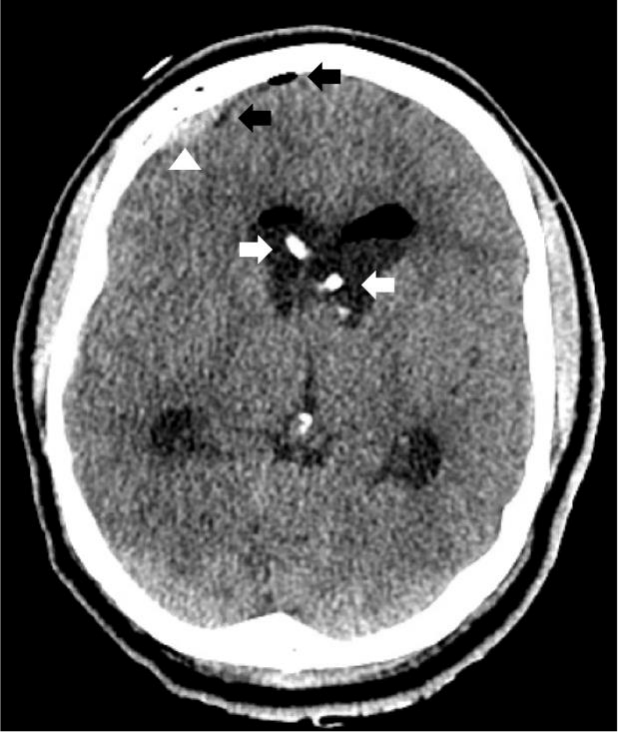
Axial computed tomography after bilateral external ventricular drains (white arrows) were placed, with post-procedural epidural hematomas (white triangle) and pneumocephalus (black arrows).

**Image 3:**
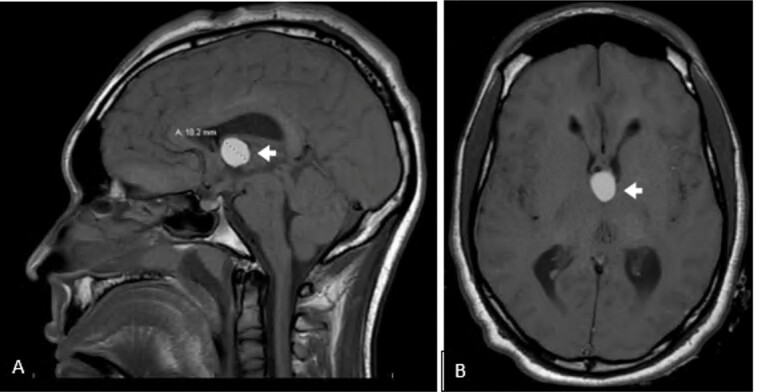
**A)** Sagittal and **B)** axial magnetic resonance image of the brain revealing 18.2-millimeter T1 hyperdense colloid cyst (white arrows) obstructing bilateral foramina of Monro.

Emergent bedside bilateral external ventricular drains (EVD) were placed (Image 2) on arrival to the accepting ED. Magnetic resonance imaging of the head revealed a colloid cyst obstructing the bilateral foramen of Monro, resulting in hydrocephalus (Image 3A and 3B). The cyst was resected via craniotomy. The patient was discharged with profound neurological disability after a prolonged inpatient stay to an inpatient rehabilitation facility. Five months after discharge, he followed commands, verbally responded to binary questions, fed himself, and stood with physical therapy assistance.

## DISCUSSION

This case highlights two pertinent points. First, it illustrates the difficulties faced by rural EPs attempting to transfer critical patients when many of the large academic referral centers or regional hospitals are on diversion and refusing transfers, and the need for improvements in facilitating timely transfers of critically ill, time-sensitive patients. Second, EPs should be aware of colloid cysts as a rare (incidence about 3.2 per million per year)^7^ but potentially catastrophic cause of rapid neurologic decline due to increased intracranial pressure (ICP), and the ED management thereof.

Interhospital transfers in the United States are regulated by the Emergency Medical Treatment and Active Labor Act (EMTALA). This act was initially intended to prevent “patient dumping” or the medically unnecessary transfer of patients for strictly financial or insurance reasons.^8^ Hospitals receiving funding from the Centers of Medicare and Medicaid Services face three obligations under EMTALA.^8^ First, they must provide a medical screening exam by qualified medical personnel to determine whether an emergency medical condition (EMC) is present. Secondly, if an EMC is present, the patient must be stabilized or transferred. Finally, referral hospitals with specialized services (such as neurologic ICUs and neurosurgery) must accept transfers of patients with EMCs without regard to financial or insurance considerations. In addition, several criteria must be met for a transfer to be “appropriate”:^9^ the transferring hospital must treat and stabilize to minimize the risk of transfer; the receiving hospital must have space and qualified personnel and agree to the transfer; the transferring facility must send all relevant documentation; and the transfer must take place through the use of qualified personnel and appropriate transportation equipment,

Several of these regulations are relevant to our case. The transferring facility and EP fulfilled all obligations under EMTALA prior to transfer, including aggressive stabilizing resuscitation and the use of critical care air transport, the highest level available. The receiving hospital did not initially have the requisite space in the neurological ICU to render the transfer “appropriate,” and the transfer call center could not agree to it. After discussion between the receiving EP and the attending neurosurgeon, space was found in the ED with plans to move the patient as quickly as possible to the neurologic ICU and neurosurgical care was available on arrival and throughout the patient’s stay in the accepting ED. With adequate space and personnel assured and agreement from the neurosurgical team, the transfer could proceed.

While this unorthodox transfer may be deemed legally “appropriate,” our patient almost died while waiting for any referral hospital to accept him in transfer. The only way the patient was treated in a timely manner was to “go around” the transfer system and directly call an EP personally known to transfer the patient from ED to ED with approval of the on-call neurosurgeon. While no individual neurosurgeon would likely refuse such a patient, the current transfer system does not allow for clinicians from external facilities to directly discuss cases and any attempts to accept transfers are often left to transfer centers, not clinicians. If the hospital is on diversion, then the transfer is denied. These transfer centers were designed with good intentions to streamline and centralize transfers to referral centers, and to help alleviate crowding at these centers by preventing “unnecessary” transfers or those for which there were not adequate resources. However, in certain cases, the transfer center pathway may exacerbate the problem faced by rural EPs. Every hospital system having a slightly different transfer pathway may also increase the difficulties faced by transferring EPs.

Rural EPs must also contend with geography during the transfer process. In many regions of the US, there may only be one academic or referral center for an entire state or even a region of several states. This fact likely increases transport time and distance. If that one center is on diversion, then the transport difficulties are even further exacerbated.

The time, effort, and frustration that occurs while attempting to get acceptance for a transfer is both demoralizing for EPs and potentially dangerous for patients. This excludes the time spent away from other active patients present in the ED, which can exacerbate already stressed and overpopulated EDs.^3^,^10^ This case highlights the difficulties that rural EPs face when not just one but six hospitals were unable to accept a transfer and the potentially fatal consequences when hospital systems are full beyond capacity. It is likely that cases such as this will continue to occur until systemwide improvements in boarding, diversion, and transfers are implemented.

Especially with prolonged transport, transfer, or boarding times, EPs may be responsible for managing patients with increased ICP for prolonged periods. Several measures may temporize patients with increased ICP until definitive neurosurgical intervention. Early consideration of intubation is critical.^11^,^12^ Adequate sedation and analgesia may control agitation and ventilator dyssynchrony, which increase ICP.^12^,^13^ Head of bed elevation with neutral neck positioning can optimize cerebral venous outflow.^11^–^13^ Optimization of blood pressure and temperature are associated with improved neurologic outcomes and mortality.^12^ Hypertension should be managed with titratable, short-acting antihypertensives, such as labetalol, nicardipine, or clevidipine, avoiding hypotension and reduction in cerebral perfusion.^12^ Normothermia (36–37.5°C) should be maintained through the use of acetaminophen or active cooling.^12^ Correction of coagulopathy may expedite neurosurgical treatment when available.

Hyperosmolar therapy may decrease cerebral edema and ICP temporarily by drawing interstitial fluid into systemic circulation.^11^,^12^ The two mainstays of treatment are mannitol and hypertonic saline.^14^ However, mannitol is contraindicated in patients with renal failure and may result in hypotension.^12^ Hypertonic saline is commonly administered as 3% sodium chloride (NaCl) 250–500 mL bolus, followed by infusion, but can be given in bolus concentrations as high as 23.4% NaCl (typical dose 30 mL) with central access.^11^

Hyperventilation can provide short-term ICP decrease due to cerebral vasoconstriction in response to hypocarbia.^12^,^13^ Effects are almost immediate but short lived, and may lead to cerebral ischemia;^11^,^12^so, hyperventilation is not recommended for routine management of ICP elevation except as an extremely short-term bridge to definitive neurosurgical treatment.

Further management of elevated ICP is dependent on the etiology. Corticosteroids can improve vasogenic edema in patients with brain tumors or abscesses, although worse outcomes are associated with steroids in traumatic brain injury and stroke.^15^,^16^ In cases of decompensated hydrocephalus, emergent diversion of cerebrospinal fluid (CSF), typically by EVD placement, is necessary to relieve ICP elevation. Bilateral EVDs can be required for third ventricular lesions that occlude the bilateral foramen of Monro,^13^ as in this case (Image 2).

Definitive management of symptomatic colloid cysts causing obstructive hydrocephalus incudes cyst removal or fenestration, permanent CSF diversion via ventriculoperitoneal shunt, or both.^17^ The Colloid Cyst Risk Score can be used to determine the risk for hydrocephalus and, therefore, the need for treatment of minimally symptomatic or asymptomatic colloid cysts.^7^

## CONCLUSION

This case illustrates the difficulties faced by rural emergency physicians when attempting to transfer critical patients when large referral centers and regional medical centers are at full capacity and refusing transfers, and the need for improvements in facilitating timely transfers of critically ill, time-sensitive patients. Additionally, EPs should be aware of colloid cysts as a rare but potentially catastrophic cause of rapid neurologic decline due to increased intercranial pressure requiring emergency neurosurgical evaluation and possible surgery. The ED management of colloid cysts and increased ICP is reviewed.
